# 3D printable model of an entomological pinning block, designed for precise positioning of entomological glue boards and labels

**DOI:** 10.3897/BDJ.12.e121569

**Published:** 2024-03-27

**Authors:** Ilia Vladimirov Gjonov, Andrey Hristozov

**Affiliations:** 1 Sofia University, Faculty of Biology, Sofia, Bulgaria Sofia University, Faculty of Biology Sofia Bulgaria; 2 N/A, Sofia, Bulgaria N/A Sofia Bulgaria

**Keywords:** entomological pinning block, label pinning, glue board pinning, entomological collections, entomological pinning gauge

## Abstract

The entomological pinning block is a device widely used by entomologists to facilitate the mounting of glue boards and labels on entomological pins. The most commonly used entomological blocks are wooden blocks with steps of varying heights, allowing the placement of individual glue boards and labels at different levels. Models of entomological blocks ready for 3D printing are scarce on the Internet. The proposed model of an entomological block is ready for printing on a standard 3D printer. In addition to the usual positioning of glue boards and labels along the Z-axis, the model also offers targeting devices that enable precise positioning of the entomological pin along the X- and Y-axes. The proposed model can be easily modified for use with glue boards or labels of a different width than proposed or with label level steps of varying heights along the pin. The design of the new pinning block is simple, yet effective, making it an accessible and useful tool for entomologists, museum curators and amateur collectors.

## Introduction

The mounting and display of insect specimens is an important part of entomological research and education. Correct mounting and positioning are essential for accurate scientific observations and comparisons. In addition, consistent placement of labels on the pin is valuable for aesthetic reasons and for optimal use of space in entomological drawers. The entomological pinning block (or gauge) is a device widely used by entomologists to facilitate the mounting of glue boards and labels on entomological pins.

Traditional entomological pinning blocks are widely used for this purpose, but they have limitations in terms of fine positioning of the needle on the label or glue board. The most traditional entomological blocks are wooden blocks with steps of different heights, allowing individual glue boards and labels to be placed at different levels. These blocks are often inaccurately machined - of the four blocks available from two different manufacturers, the depth of the holes for label positioning along the Z-axis of the same step varies by up to 1 mm and in the height of the steps by up to 1.4 mm. Steel entomological pinning blocks are precise in z-positioning, but are relatively expensive and rarely sold (https://www.entosupplies.com.au/equipment/laboratory/pinning-labelling/pinning-blocks/; https://ecologysupplies.com/products/metal-pinning-block/ and https://www.watdon.co.uk/acatalog/E700-pinning-stage.html). The majority of the 3D printing models on offer are only used to position the label or the glue board along the Z-axis (https://www.thingiverse.com/thing:737677; https://www.thingiverse.com/thing:4686188; https://www.thingiverse.com/thing:2423546 and https://www.printables.com/model/457221-entoblock). One model (https://www.thingiverse.com/thing:260983) provides X- and Y- positioning, but no Z-axis positioning and no pin target mechanism. So far, only Ghafouri Moghaddam et al. (2017) have proposed a model that suggests positioning along the three spatial axes. The elements of this model are shown in the publication and it is intended to be made from Plexiglas. It is relatively difficult to make and requires precision tools, but it can be easily modelled for 3D printing. Its use seems practical and efficient for applying labels, but it is rather complicated and does not allow the use of different widths of glue boards. A detailed analysis of the different types of entomological pinning blocks can be found in Ghafouri Moghaddam et al. (2017), who propose their own practical design for the uniform application of multiple labels of the same size.

In this paper, we present a new 3-dimensional model of an entomological pinning block that allows for more precise pin positioning. The design of the new pinning block was inspired by the need for a more precise and efficient mounting tool.

## Materials and Methods

The free 3D modelling software Blender v. 4.0.2 (GNU GPL version 3 licence) (Blender Online Community 2018) was used to create the model. The model was sliced for 3D printing using UltiMaker Cura v. 5.6.0 software (GNU GPLv3 licence) (Ultimaker B.V. 2023) and successfully printed multiple times on a Creality Ender-3 Pro 3D printer using both PLA and PETG materials with stable results.

## Description

The proposed model of an entomological block is ready for printing on a standard 3D printer.

The five positioning steps along the Z-axis of the proposed model are the same - exactly 5 mm. They can be parameterised if required. In addition to the usual positioning of glue boards and labels along the Z-axis, the model also provides grooves and cut-outs that allow the entomological pin to be positioned precisely along the X- and Y-axes. Holes of 1 × 1 mm and 2 × 2 mm are provided so that the normally stiff cardboard can easily be pierced with the pin in the required position.

The model is designed for glue board or label widths of 4 to 10 millimetres and, when working correctly, the needle is inserted exactly in the centre of the board, 1 millimetre from the back. These widths and distances can also be parameterised by the user.

**Figure 1.**
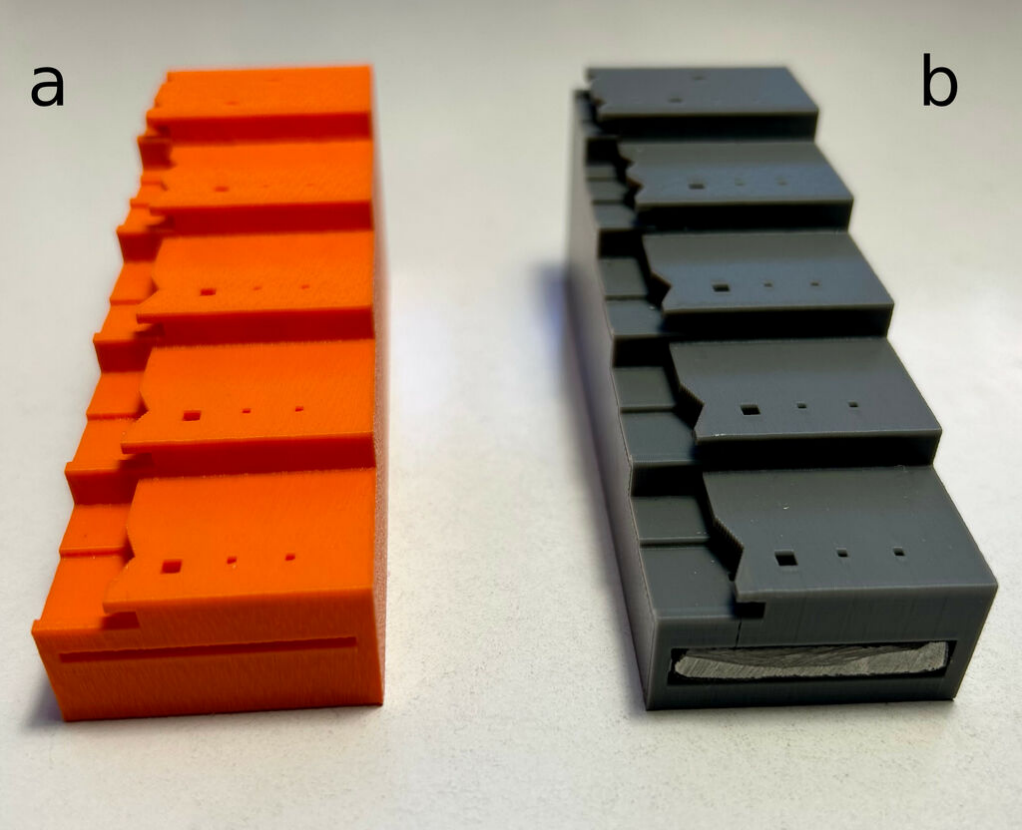
3D entomological pinning block in two versions: **a** without steel rail; **b** with steel rail.

**Figure 2.**
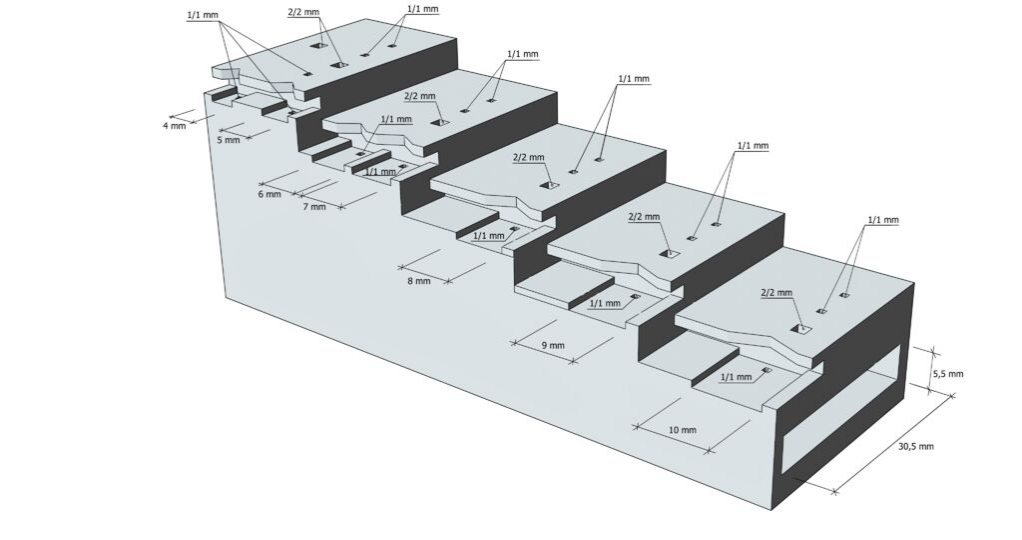
Dimensions of the entomological pinning block with metal rail.

The model is offered in two variants - one for immediate use after printing (Fig. 1a; Suppl. material 1) and another that requires the fabrication of an additional steel rail measuring 100 × 30 × 5 mm, against which the tip of the pin rests during positioning along the Z-axis (Fig. 1b; Suppl. materials 2, 3). The rail is also used to increase the overall mass of the fixture. The overall dimensions of the device are 100 mm in length, 35 mm in width and 37 mm in height (Fig. 2).

When printed with polylactic acid (PLA) or polyethylene terephthalate glycol (PETG), the device has a mass of 40 to 50 grams, depending on the slicer settings. PLA is the most commonly used material for 3D printing. It is relatively chemically resistant, but dissolves easily in chloroform, has relatively low strength and cannot withstand very high temperatures. PETG, on the other hand, is stronger, less soluble and can withstand relatively high temperatures, but is more difficult to print.

The model was successfully printed using the Creality Ender 3 Pro 3D printer with the following parameters:

- Nozzle diameter: 0.4 mm
- Layer height: 0.2 mm
- Wall thickness: 0.8 mm
- Top/bottom thickness: 0.8 mm
- Infill Density: 20%
- Infill pattern: Cubic
- Printing temperature: PLA - 210°C; PETG - 230°C
- Build plate temperature: PLA - 60°C; PETG - 80°C
- Cooling: 40%
- Support: none

Printing time is approximately 5 hours.

With a PLA/PETG price of around €15 per kg, the calculated cost of the material is €0.60-0.65 per unit. When using a 3D printing service, prices vary between €0.05 and €0.25 per gram, so the cost of 3D printing a model can range from €2.5 to €12.5.

## Implementation

The proposed entomological block is intended for widespread use by entomologists, particularly those using insect glue boards. It is applicable to large entomological collections with individual specimen numbering and its use can ensure that entomological pins are positioned on the label so as not to disrupt the integrity of the number or barcode. It can also be modified to suit the needs of the user and can be sliced and printed directly on a 3D printer.

### Methodology

**Figure 3.**
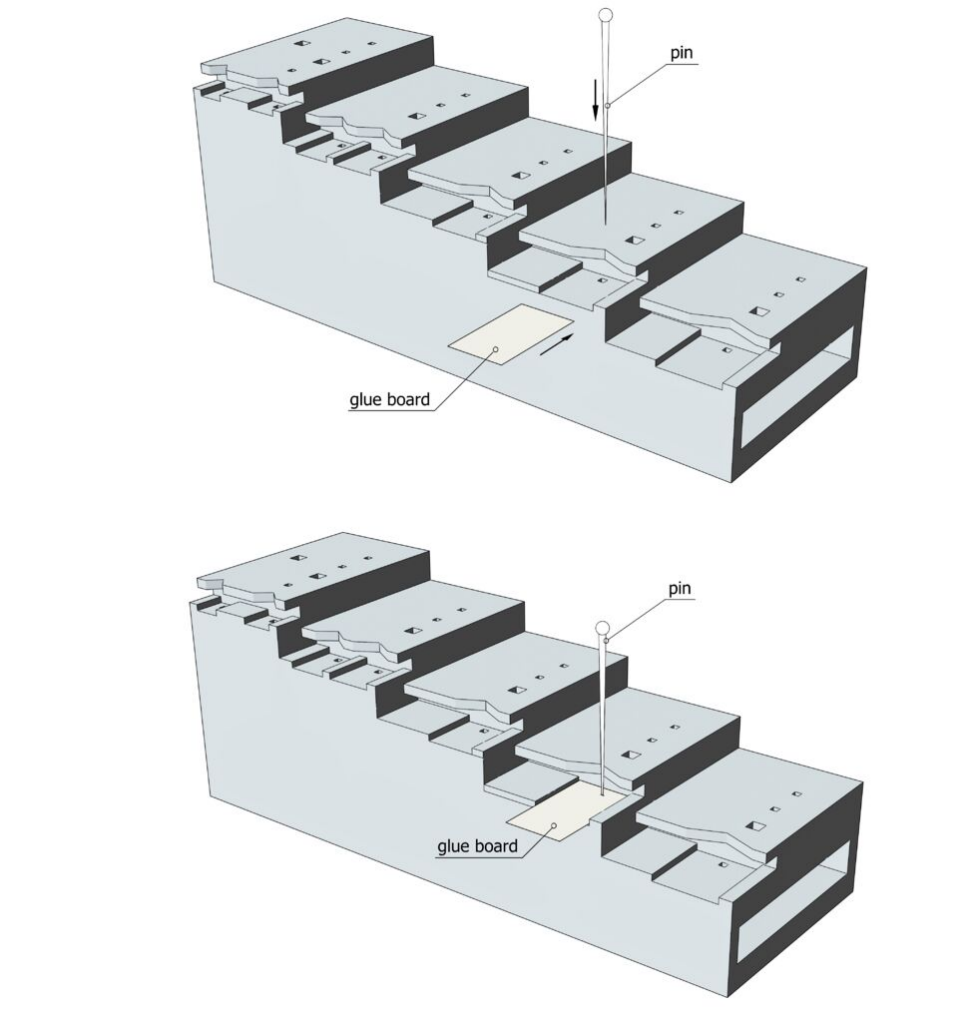
Operating mode of X- and Y-positioning for the entomological pinning block.

The first step is to use the X and Y positioning slots, by inserting the label or card into the appropriate slot, pushing it all the way in and piercing it at right angles with the needle positioned in the special angled cut-out. The label, drilled in the correct position, is then positioned along the Z-axis by pushing the needle into one of the holes on the corresponding step. The wide 2 × 2 mm hole can be used for wider labels or cartons, while the narrow 1 × 1 mm hole is recommended for narrower labels (Fig. 3; Suppl. material 3).

## Re-use potential

The proposed model can be easily modified for use with glue boards or labels of a different width than proposed or with label level steps of varying heights along the pin.

## Conclusions

The new pinning block offers a significant improvement over traditional pinning blocks, which often lack the fine positioning capabilities required for accurate mounting and observation. The design of the new pinning block is simple, yet effective, making it an accessible and useful tool for entomologists, museum curators and amateur collectors. This new model will be a valuable addition to the entomological toolkit and will assist in the advancement of entomological research and education. It can be easily parameterised to the user's requirements prior to printing to meet a wide range of insect labelling needs.

## Supplementary Material

E18A05E4-9F89-5ED0-9213-C27131BA221F 10.3897/BDJ.12.e121569.suppl1 Supplementary material 1 Entomological pinning block without a metal rail Data type stl 3D model Brief description 3D model of the entomological pinning block ready to use immediately after printing. File: oo_981345.stl https://binary.pensoft.net/file/981345 Ilia Gjonov and Andrey Hristozov

6849CD13-19AE-53BF-A5A8-6FD721BA7421 10.3897/BDJ.12.e121569.suppl2 Supplementary material 2 3D model of the entomological pinning block with a metal rail Data type stl 3D model Brief description 3D model of the entomological pinning block suitable for finishing by adding a metal rail. File: oo_981346.stl https://binary.pensoft.net/file/981346 Ilia Gjonov and Andrey Hristozov

8E499C57-41CD-53C0-8E07-DD9CBCCC4736 10.3897/BDJ.12.e121569.suppl3 Supplementary material 3 3D entomological pinning block demonstration video Data type mp4 video Brief description Demonstration of how to use the 3D entomological pinning block and how to assemble the steel rail version. File: oo_990979.mp4 https://binary.pensoft.net/file/990979 Ilia Gjonov and Andrey Hristozov
